# Bone Marrow Stromal and Vascular Smooth Muscle Cells Have Chemosensory Capacity via Bitter Taste Receptor Expression

**DOI:** 10.1371/journal.pone.0058945

**Published:** 2013-03-08

**Authors:** Troy C. Lund, Amanda J. Kobs, Ashley Kramer, Mick Nyquist, Marcos T. Kuroki, John Osborn, Diane S. Lidke, Shalini T. Low-Nam, Bruce R. Blazar, Jakub Tolar

**Affiliations:** 1 Division of Pediatric Blood and Marrow Transplant, University of Minnesota, Minneapolis, Minnesota, United States of America; 2 Department of Integrative Biology and Physiology, University of Minnesota, Minneapolis, Minnesota, United States of America; 3 Department of Pathology and Cancer Research and Treatment Center, University of New Mexico, Albuquerque, New Mexico, United States of America; University of Udine, Italy

## Abstract

The ability of cells to detect changes in the microenvironment is important in cell signaling and responsiveness to environmental fluctuations. Our interest is in understanding how human bone marrow stromal-derived cells (MSC) and their relatives, vascular smooth muscle cells (VSMC), interact with their environment through novel receptors. We found, through a proteomics screen, that MSC express the bitter taste receptor, TAS2R46, a protein more typically localized to the taste bud. Expression was also confirmed in VSMCs. A prototypical bitter compound that binds to the bitter taste receptor class, denatonium, increased intracellular calcium release and decreased cAMP levels as well as increased the extracellular release of ATP in human MSC. Denatonium also bound and activated rodent VSMC with a change in morphology upon compound exposure. Finally, rodents given denatonium in vivo had a significant drop in blood pressure indicating a vasodilator response. This is the first description of chemosensory detection by MSC and VSMCs via a taste receptor. These data open a new avenue of research into discovering novel compounds that operate through taste receptors expressed by cells in the marrow and vascular microenvironments.

## Introduction

There are a variety of receptor types through which cells respond to changes in their microenvironment including those for cytokines, hormones, mechanical tension, and there are thousands of different receptors expressed on any given cell type [1]. It is not completely understood how mesenchymal stromal cells (MSC) and one of their proposed derivatives, vascular smooth muscle cells (VSMC), interpret and respond to their microenvironment. To discover new and novel cell surface receptors, we performed a large mass-spectrometry based proteomic screen for new and novel cell surface receptors; we found the expression of a bitter taste receptor, TAS2R46, on the surface of human MSC and confirmed expression also on VSMC.

Bitter taste receptors are typical G-protein coupled receptors (GPR) and are normally found on the surface of the tongue [2]–[4]. The human bitter taste receptor class (referred to as T2R) has over 20 members [5]. The expression of T2Rs outside of the tongue and alimentary tract has been only recently discovered as T2Rs have been shown to be expressed in airway smooth muscle cells and cause significant airway dilation/relaxation upon activation [6]. Also, T2R expression on human airway epithelial cells was specifically localized to motile cilia, and upon treatment with bitter compounds, the epithelial cells showed an increase in ciliairy beat [7]. The evolutionary explanation for bitter taste receptor expression outside the tongue is not known, but it is speculated that these receptors could serve to participate in a mechanism for clearance of noxious compounds, which are often bitter when tested in “taste” studies and directly engage the T2R receptor class.

Our data extends the prior findings of extra-gustatory taste receptor expression whereby we show bitter taste receptor expression on MSCs and VSMCs. Engagement of these receptors caused intracellular calcium release which is one of the main second messenger signaling pathways utilized by the T2R class [8]. VSMCs produced a physiological response that led to a change in morphology of cell shape and size. Rats exposed to the prototypical bitter compound denatonium, known interact with TAS2R46, had a transient and significant drop in blood pressure followed by recovery, providing in vivo evidence that a bitter compound can modulate vascular tone through engagement of its receptor.

## Results

**Figure 1:**
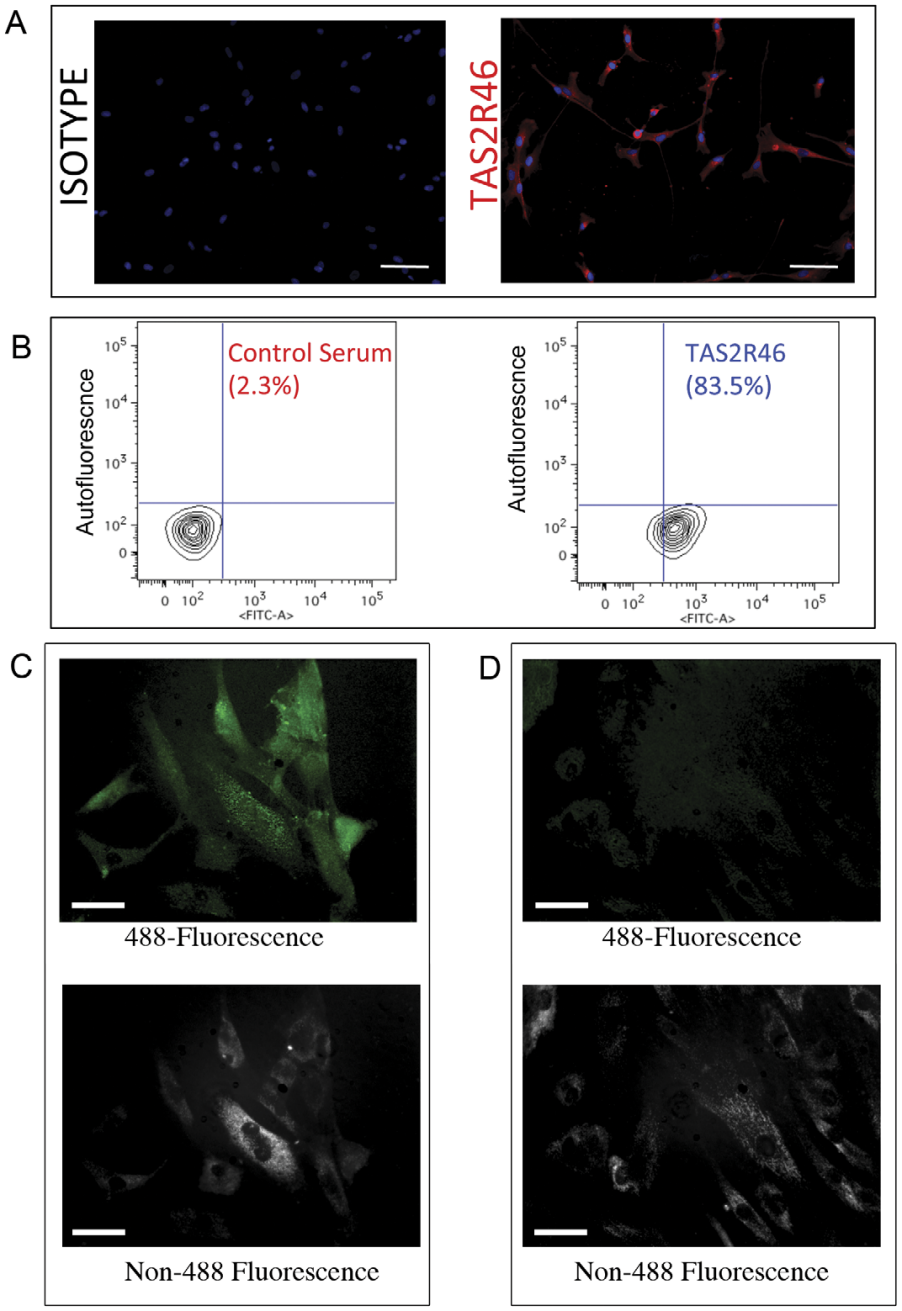
Human MSC express TAS2R46. (A) Human bone marrow-derived MSC grown in culture for 3 weeks were fixed and stained with anti-hTAS2R46 followed by Cy3-conjugated anti-rabbit secondary antibody and imaged via confocal microscopy. Scale bars represent 50 microns. (B) Flow cytometry of human MSC for hTAS2R46 using rabbit anti-hTAS2R46 (or control serum) and FITC-conjugated anti-rabbit secondary antibody. (C) Human MSC bind denatonium. Binding to MSC is visualized by Alexa-Fluor® 488 Click labeling of a DIBO-conjugated denatonium (see methods). Images were acquired on a Nuance spectral camera, permitting separation of Alexa-Fluor® 488 fluorescence (top panel) from cellular autofluorescence (bottom panel). (D) MSC labeled with Click reaction in the absence of DIBO-denatonium as a negative control shows the absence of Alexa-Fluor® 488 signal (upper panel). Cellular autofluorescence (lower panel) shows cells present in the field of view. Scale bars represent 20 microns in C and D.

**Figure 2:**
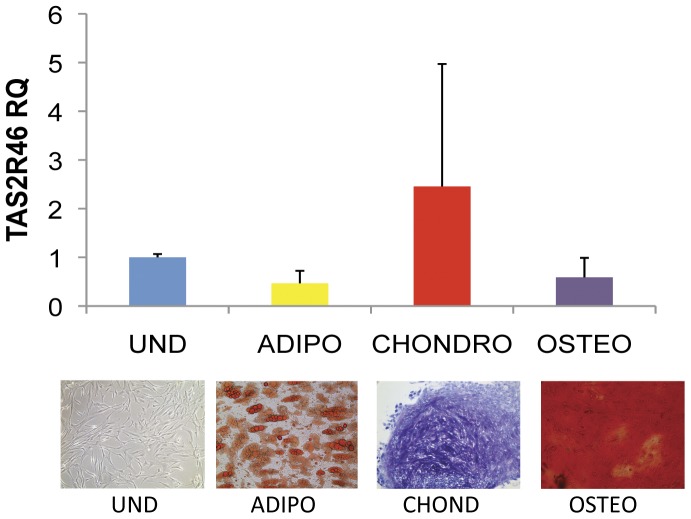
QRT-PCR for TAS2R46 on human MSC after differentiation into the adipocyte, osteocyte, and chondrocyte lineages, n = 3 donors with 3 technical replicates each. A Student's t-test showed no significant difference between each differentiated hMSC culture and undifferentiated hMSC for TAS2R46 (p-value >0.05 for each pairwise comparison). The photomicrographs below are differentiated hMSC stained for the indicated lineage. Differentiation and staining was performed as described in the methods section. The undifferentiated (UND) hMSC and differentiated images were taken under phase-contrast or bright field respectively (10× objective).

In a search to discover new and novel receptors on MSC, we subjected hMSC to an iTRAQ-based mass spectrometry analysis and discovered, to our surprise, the expression of a bitter taste receptor previously found only on the tongue known as TAS2R46 (data not shown). Because no class of taste receptor had previously been known to be expressed on MSC, we verified the unique expression of TAS2R46 by several different methods. As shown in Fig. 1, immunofluorescence assays and flow cytometry demonstrated that TAS2R46 is expressed on most hMSC previously isolated from the marrow of healthy donors. We detected no differences in TAS2R46 expression in MSC from different ages/sex of donors (data not shown). We next fluorescently labeled a prototypical bitter ligand for TAS2R46, a quaternary amine known as denatonium, using Click-iT based chemistry [3], [9]. We were able to confirm direct binding of denatonium to hMSC as shown in Figure 1C. We next used QRT-PCR to determine if *TAS2R46* gene expression was different upon differentiation of MSC into the classic mesodermal tissues of adipocytes, chondrocytes, and osteocytes and found no significant change in the amount of *TAS2R46* expression (Fig. 2).

**Figure 3:**
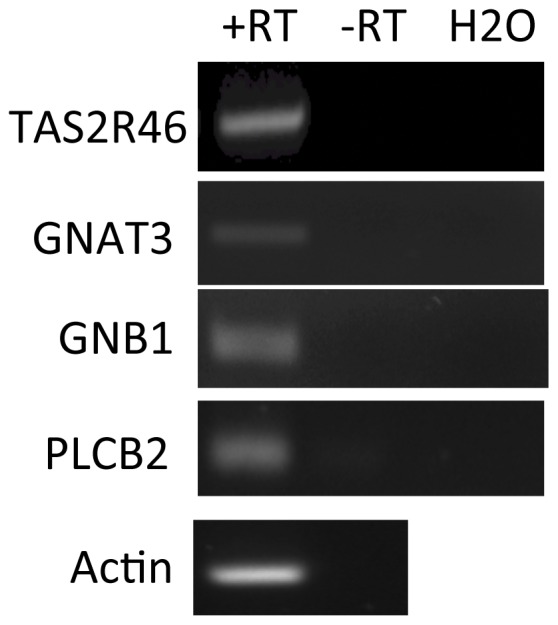
Human MSC express components of the taste receptor signaling pathway. RT-PCR was performed on hMSC from normal healthy donors approximately 3 weeks after isolation. –RT lanes are reactions without reverse transcriptase. Shown are the RT-PCR products separated and ethidium bromide stained on a 1.5% agarose gel.

As GPRs, bitter TAS2Rs work in a complex with other proteins to mediate their downstream signals via second messengers. Gustducin, a heterotrimer of alpha, beta, and gamma subunits (GNAT3, GNB1, GNG13), and phospholipase C beta 2 (PLCB2) have been shown to play a role in the signal transduction of bitter taste receptors [2], [10], [11]. RT-PCR of hMSC showed the expression of the alpha and beta subunits of gustducin (*GNAT3*, *GNB1*) as well as *PLCB2* indicating that some of necessary factors were present for signal transduction by TAS2R46 are expressed by hMSC (Fig. 3).

**Figure 4:**
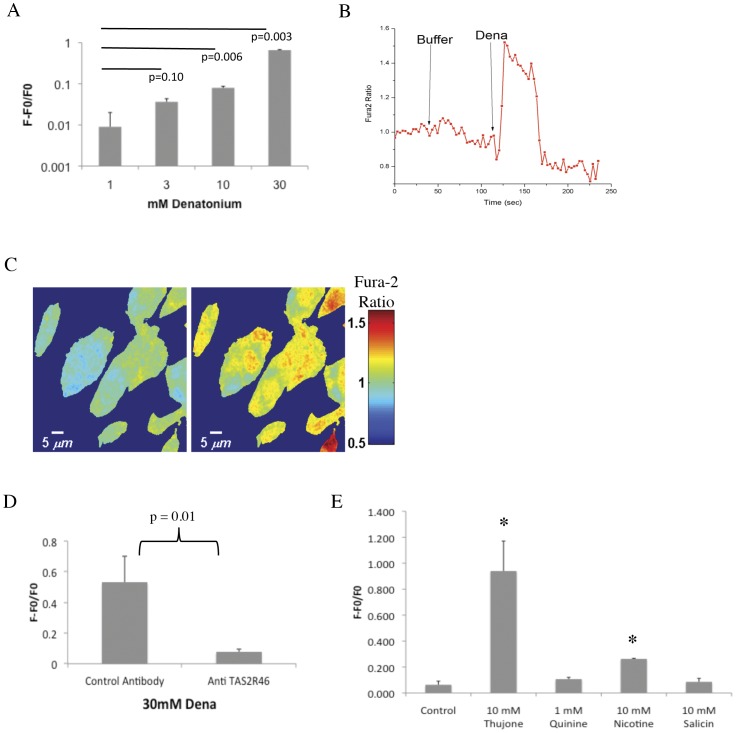
Human MSC respond to bitter compounds by increased calcium activity. (A) Human MSC were exposed to increasing amounts of denatonium followed by measurement of calcium activity at 5 minutes of drug exposure using a Fluo-4 Direct™ Calcium Assay Kit and data is displayed as increase in fluorescence over baseline. Shown are the means ± standard deviation; p-values derived from a Student's t-test. (B) Calcium release assay performed with MSC loaded with Fura2 and exposed to 1 mM denatonium. Shown is a typical response from a single cell. (C) Images represent Fura2 fluorescence after background subtraction pre and post denatonium exposure. The F/F0 is encoded by pseudocolor. (D) Denatonium stimulated calcium activity performed on hMSC with the addition of anti-hTAS2R46 antibody to block denatonium signaling. Shown are the means ± standard deviation; the p-value is derived from a Student's t-test; n = 3 experiments. (E) Human MSC calcium activity in response to other bitter compounds using a Fluo-4 Direct™ Calcium Assay Kit, Shown are the means ± standard deviation; p<0.0001 from a one-way ANOVA. The * represents p-value <0.05 in comparison to the control group from a Bonferroni post test (n = 4 experiments).

We next sought to determine the functionality of TAS2R46 by application of bitter compounds to hMSC and evaluating for calcium activity which is a key second messenger for taste receptor signaling [2]. A rapid increase in calcium activity was seen in response to denatonium when tested on hMSC using two approaches to measure calcium release: (1) a Fluoru-4 Direct™ Calcium Assay (Fig. 4A), and (2) Fura2 loaded hMSC (Fig. 4B and 4C). Specificity of a TAS2R46 mediated intracellular calcium activity was shown when we pretreated hMSC with an antibody to TAS2R46 which attenuated the denatonium induced calcium release (Fig. 4D). Other bitter compounds also known to interact with the T2R class of bitter receptors were tested including thujone, quinine, nicotine, and salicin. We found that thujone and nicotine elicited a significant calcium release response (Fig. 4E). These data show that hMSC express a functional TAS2R46 and can respond to bitter compounds, although there are two caveats worth mentioning. It is possible that other TAS2R family members could be present that respond to bitter tastants. Secondly, hMSC have been shown to express nicotinic receptors which also signal through calcium pathways and may account for the results we observe [12]. Less is known about the native signaling pathway triggered by thujone, but it is thought to engage gamma-aminobutyric acid (GABA)-type receptors that may also be present at low level on MSC, although this is not well studied [13].

**Figure 5:**
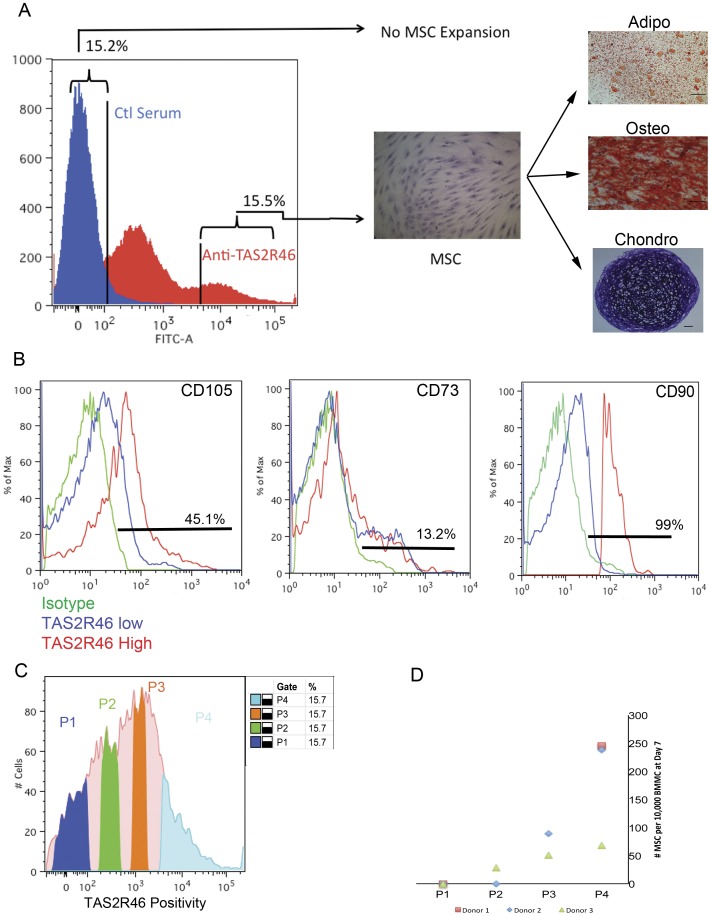
Flow cytometry sorting of TAS2R46 positive human bone marrow mononuclear cells. (A) Approximately 10 milliliters of fresh human bone marrow was subject to Ficoll separation. The mononuclear cells were stained with rabbit anti-TAS2R46 and appropriate secondary antibody. TAS2R46 high sorted cells (roughly 705,000 cells) were placed into hMSC growth medium and incubated at 37C, 5% O_2_, 5% CO_2_. The resulting hMSC culture from donor 1 expanded within 1 week post-sort and shown in A. The TAS2R46 negative/low fraction did not result in any hMSC outgrowths. Crystal violet staining of hMSC was performed 2 weeks after isolation. Tri-differentiations were performed three weeks after isolation and stained appropriately. Scale bars represent 100 microns. (B) Bone marrow mononuclear cells were isolated and costained for TAS2R46 and classical markers of MSC: CD105, CD90, and CD73. Expression of each of these markers is shown with respect to prior gating on TAS2R46 high versus TAS2R46 negative/low expression. (C) Example of sorting strategy into populations of high to low TAS2R46 expression for two other bone BMMC donors. (D) Enumeration of MSC counted at day 7 of culture expansion normalized to the input number of sorted BMMC. Note P1 of all three donors gave rise to no MSC.

To determine whether MSC could be isolated *a priori* based on the expression of TAS2R46 from fresh bone marrow mononuclear cells (BMMC), flow cytometry-based sorting was performed on freshly harvested human BMMC. Most of the MSC potential was confined to the TAS2R46^high^ fraction, while no MSC expanded from the TAS2R46^low^ cells (Fig. 5A). In a separate experiment, we costained for TAS2R46 and we the classical markers for MSC: CD105, CD73, and CD90. We found that TAS2R46^high^ cells displayed much higher levels of these markers than TAS2R46 low/negative cells (Fig. 5B). Secondarily, for each of the MSC markers, if we gated for CD45 negative cells (typical of MSC), we found that TAS2R46high/MSC marker positive/CD45^negative^ cells represented 0.001–0.03% of bone marrow mononuclear cells (data not shown) which is in the range predicted for in vivo MSC [14].

We employed and additional approach in which we labeled hBMMC with anti-TAS2R46 and flow-sorted based not only on the highest and lowest 15%-ile TAS2R26 expressing cells (as in Fig. 5A), but also on two intermediate staining populations as well – encompassing the whole range of TAS2R46 expression (strategy shown in Fig. 5C). We enumerated the hMSC resulting from expansion cultures of the sorted cells after 7 days of growth and normalized to the number of input BMMCs. We found the highest number of MSC (50–250 MSC per 10,000 BMMC) came from the BMMCs expressing the greatest amount of TAS2R46 (the P4 population) as shown in Fig. 5D. Again, we found that the lowest TAS2R46 expressing population (P1) gave rise to no MSC. Intermediate TAS2R46 expressing BMMC (P2 and P3) gave rise to lesser numbers of MSC. All MSC derived were negative for CD31, CD34, CD45, and positive for CD73, CD90, and CD105 expression as is typical of hMSC (Fig. S2). The P4-derived hMSC were differentiated into the classic MSC lineages of adipocytes, osteocytes, and chondrocytes followed by appropriate staining for these lineages as exampled in Fig. 5A. We noticed no differences in differentiation ability in any hMSC line derived (data not shown).

These data show that TAS2R46 can be used effectively to isolate cells from the bone marrow with MSC potential and greater TAS2R46 expression correlates to increased numbers of MSC in expansion cultures. It is unknown how efficient TAS2R46 is as an MSC marker because TAS2R46 expression is somewhat variable from cell to cell as evidenced by the earlier immunofluorescence studies and also our cell culture conditions favor the outgrowth of only MSC. The in vivo identity of hMSC remains unknown, but some have speculated they originate as perivascular cells which may be related to vascular smooth muscle cells which we investigate later [15].

The downstream effects of T2R signaling are not well understood. In addition to calcium as a second messenger, investigators have determined that bitter taste receptors can also decrease levels of cAMP [10]. We found that after denatonium treatment, hMSC cAMP levels decreased by 40% (Fig. S3) similar to what has been described previously in taste buds [10]. In addition to decreasing cAMP, engagement of the bitter taste receptors has also been shown to increase extracellular ATP release [16] which we also found to be true in hMSC after exposure to denatonium (Fig. S3) These data show that hMSC respond to denatonium with several secondary signals known to be involved in taste transduction.

**Figure 6:**
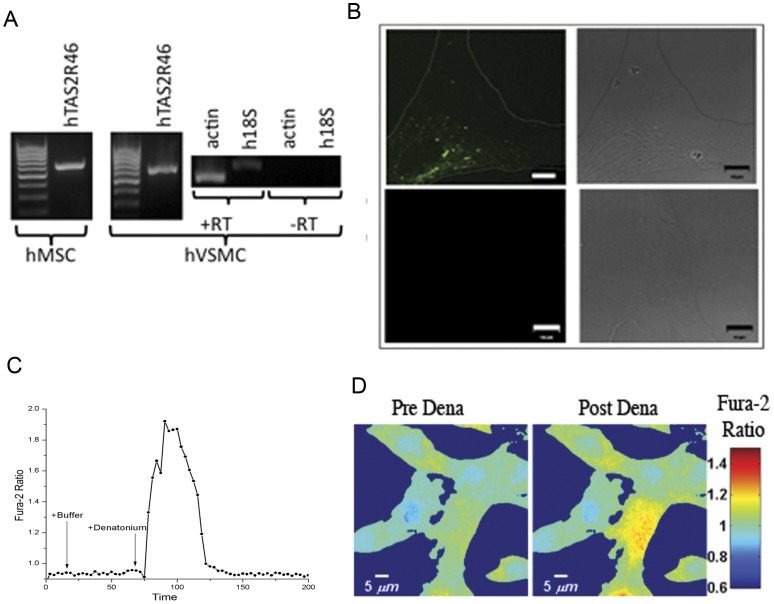
Human vascular smooth muscle cells express hTAS2R46 and are stimulated by denatonium. (A) RT-PCR of hTAS2R46 in cultured hVSMCs. (B) Human VSMC stained with denatonium which had undergone direct conjugation to Alexa-Fluor-488 using Click-iT® amine DIBO alkyne chemistry. Top panels show conjugated denatonium and bottom panels show staining with Click-iT® reaction solution alone. Scale bars represent 10 microns. (C) Calcium activity assay performed with hVSMC loaded with Fura2 and exposed to 1 mM denatonium. Shown is a typical response from a single cell (of greater than 10 cells tested). (D) Images represent Fura2 fluorescence in response to denatonium treatment after background subtraction. The Fura-2 ratio is encoded by pseudocolor. Note that not all cells responded equally in the same time frame.

Another mesodermal derived cell type is the vascular smooth muscle cell. It has been hypothesized that MSC may be an ancestor to VSMCs [17]. VSMCs then play a key role in the maintenance of vascular tone and integrity. We assessed human VSMCs for expression of TAS2R46 by RT-PCR which was positive (Fig. 6A). We also found human VSMCs capable of binding to fluorescently labeled denatonium (Fig. 6B). Non-quantified imaging suggested that the binding was not as robust as with hMSC because the fluorescent signal intensity was decreased on hVSCMC compared to hMSC. We were also able to demonstrate that hVSMCs released intracellular calcium when exposed to denatonium in vitro as with hMSC (Fig. 6C, D).

Demonstrating that hVSMCs expressed a bitter taste receptor and responded to denatonium led us to believe that engagement of this receptor may produce a vascular effect. The gustatory cells of rodents are well-known to express bitter taste receptor class [9]. We evaluated two of the known bitter taste receptors, TAS2R116 and TAS2R143, on mouse VSMCs by RT-PCR and found they were expressed (Fig. S4). We noticed that some mouse VSMCs showed a significant change in morphology after exposure to denatonium (Fig. S4). The mechanism of this morphological change is not known as of yet, but the cells appeared to adopt a relaxed or expanded phenotype.

**Figure 7:**
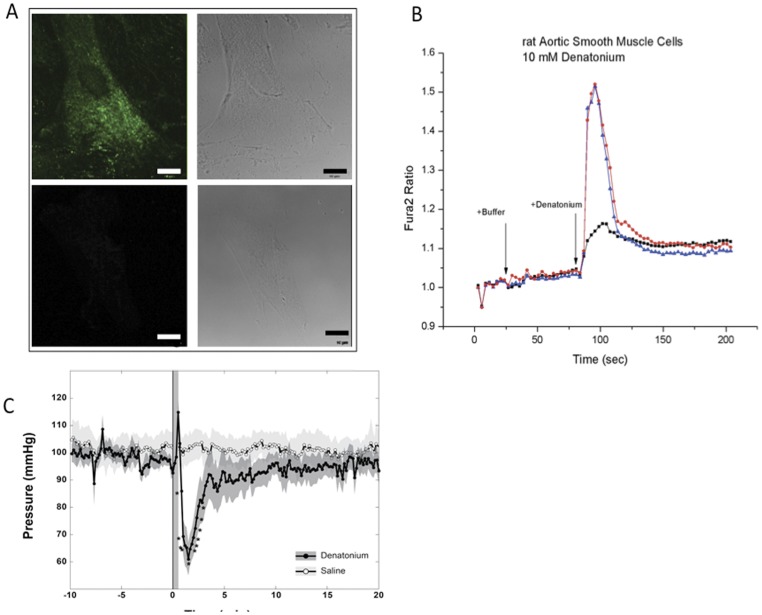
Rat vascular smooth muscle cells respond to denatonium. (A) Rat VSMCs bound denatonium which had undergone direct conjugation to Alexa-Fluor-488 using Click-iT® amine DIBO alkyne chemistry. Top panels show conjugated denatonium and bottom panels show staining with Click-iT® reaction solution alone. Scale bars represent 10 microns. (B) Calcium release assays were performed with rVSMC loaded with Fura2 and exposed to 10 mM denatonium. Shown is a typical response from two separate cells. (C) Sprague Dawley rats underwent inferior vasa cava cannulation with blood pressure transducers. One micromole of denatonium was delivered via tail vein injection with blood pressure monitoring for 20 minutes post injection. Control rats received saline of an identical bolus volume. Data shown represents results from n = 6 rats per group.

Finding bitter taste receptor expression and the change in morphology in mouse VSMCs combined a recent report that airway smooth muscle cells express bitter taste receptors contributed to bronchodilation [6], suggested to us that engagement of bitter taste receptor could cause an effect on blood pressure. Because rat models of vascular tone are more practical, we also analyzed rat VSMCs, which we found to bind to labeled denatonium (Fig. 7A). Rat VSMCs also responded to denatonium by releasing intracellular calcium as shown by Fura2 calcium flux imaging similarly as in the prior experiments (Fig. 7B). To test the effect of denatonium in vivo, 1 micromole of denatonium was injected into Sprague Dawley rats cannulated with blood pressure transducers and monitored for vascular response. Blood pressure decreased significantly within 1 minute of denatonium exposure and returned to baseline over the next two to five minutes (n = 5 rats tested) (Fig. 7C). This hypotensive response supports the hypothesis that denatonium acts though bitter taste receptors on VSMCs, initiating a relaxed state leading to vasodilation.

## Discussion

Our findings describe the first ever expression and physiological function of a taste receptor in both MSC and VSMCs. This receptor class represents a new means for these cell types to interact with their microenvironment. Until recently, taste receptors have not been described outside the gustatory or alimentary systems. Two recent papers describe the expression of bitter taste receptors in airway epithelial cells and airway smooth muscle cells [6], [7]. The description of bitter taste receptors on ciliated epithelial cells represents the first report of the bitter taste receptor in the respiratory system. It was shown that engagement of the bitter receptors increased intracellular calcium as well as increased the beat time of cilia. This action is hypothesized to be a response to “clear” potentially toxic compounds from the airway which are often bitter [18]. Bitter receptors have also recently been shown to be present on airway smooth muscle cells and can efficiently act as a bronchodilator when engaged by bitter compounds. The response was not altered by nitric oxide synthase inhibition and was therefore assumed to be a direct result of the bitter compounds. In contrast to our data, this same study showed that mouse trachea relaxation was associated with an increase in cAMP, while our data showed a decrease in cAMP which is consistent with that found in taste tissue [10]. The finding of bitter taste receptors on air smooth muscle cells led to further speculation that expression of this receptor class in extra-gustatory tissues is protective against worsening pneumonia caused by gram-negative organisms as they secrete acyl-homoserine lactones which have been previously shown to engage bitter receptors although this has not been proven [6].

The precise relationship between MSC TAS2R expression and that of VSMC is unclear. There is some literature pointing toward MSC being an ancestral cell to the VSMC [19], and our data show that bitter taste receptor is present on both cell types illustrating that TAS2R expression is more widespread than previously recognized, although we did recognize a different, though not quantified, distribution in TAS2R receptor numbers in our denatonium binding experiments depending on the cell type (data not shown) leading one to believe that there are differences in TAS2R receptor regulation. We found that TAS2R46 is expressed on freshly isolated bone marrow mononuclear cells from which MSC can be derived, indicating de novo expression of TAS2R46 rather than acquired expression during hMSC culture expansion. We also find that TAS2R46-high expression is shared with classical markers of MSC (CD105, CD90, and CD73). Similar to prior reports, our findings indicate that engagement of the bitter receptor causes an increase in intracellular calcium release that can function as a secondary messenger in MSC and VSMC. Our data also showed a decrease in cAMP levels and increase in extracellular ATP measured in MSC, consistent with gustatory cell signaling, though other signaling pathways remain to be resolved [10], [20].

To our knowledge, these are the first experiments to deliver denatonium systemically and witness as physiologic response. There is some thought that vertebrates have evolved bitter taste receptors to respond to, and avoid, noxious plants and foods. Our studies in the rat model to assess physiological effects of denatonium showed a rapid and transient drop in blood pressure. This could be perceived as a “noxious” response. Additionally, the “therapeutic window” of the denatonium response was narrow as amounts over 1 micromole caused severe hypotension and death in the animals. We hypothesize that it is through engagement of TAS2R on the VSMCs that this response is occurring, although direct in vivo engagement would be difficult to show. Another explanation for our observed effect on blood pressure could be the engagement of additional receptors such as adrenergic receptors, especially considering the higher doses of denatonium used in our in vitro experiments. Interestingly, Zhang et al has recently shown that a variety of adrenergic receptors are found on taste bud cells from rats, although specific engagement has yet to be shown [21]. A speculative interaction between taste receptors and adrenergic receptors would be interesting and has yet to be explored. Future studies using an in vivo blocking agent to TASR2s will be useful, but such an antagonist has yet to be identified.

As to another teleological answer of why there is expression of a bitter taste receptor in the vasculature, it seems possible that this receptor exists to engage a hormone or small peptide. In an initial attempt to explore potential peptide engagement of TAS2R46, we utilized casein hydrosylate, which is known to contain a large number of bioactive peptides [22], applied to TAS2R46-overexpressing hMSC and found increased calcium release from intracellular stores (Fig. S5). We are now attempting to identify the specific bioactive peptide responsible for this response. Additional support of a peptide being an in vivo ligand for T2Rs come from prior studies showing that certain dipeptides are bitter in taste quality and can engage bitter taste receptors resulting in increased intracellular calcium [22], [23]. Finally, but somewhat contrary to the peptide ligand theory, foods such as teas and chocolate are known to lower blood pressure. These foods also contain high concentrations of bitter compounds such as flavanols; leading to the speculation that the lower vascular tone associated with consumption of these foods is driven by bitter taste receptor engagement [24]–[26]. Overall, our data show the expression of taste receptors is much more extensive than previously appreciated and includes MSC and VSMCs as TAS2R expressing cell types. These observations open a new area for fertile research in discovering the cognate ligands and various biologic in vivo role of this unique receptor class.

## Materials and Methods

### Ethics Statement

All studies on human cells were approved by the Committee on the Use of Human Subjects in Research at the University of Minnesota. All of the animal studies were conducted in accordance with the National Institutes of Health Guide for the Care and Use of Laboratory Animals and approved by the University of Minnesota Institutional Animal Care and Use Committee.

### Cell Isolation/Culture

Human mesenchymal stromal cells were isolated and maintained as previously described [27]. A variety of hMSC lines were used from six donors ages 1–19 years old. We found no difference in TAS2R46 expression or response to denatonium with regard to age or sex of the donor (data not shown). hMSC were used prior to their 4^th^ passage.

Human aortic smooth muscle cells were purchased from ATCC (PCS-100-012) and grown in Medium 231 (Life Technologies, Grand Island, NY) with smooth muscle differentiation supplement (Life Technologies, #S-008-5) and 1 ng/ml of recombinant Human TGF-beta 1 (R & D systems, #240-B-002).

Isolation of mouse and rat vascular smooth muscle cells was based on the method of Ray et al [28]. Mice were of strain FVB or C57BL/6. Mice were sacrificed using carbon dioxide and dissection performed in a designated mammalian dissection hood. The aorta was harvested and placed into sterile PBS. Most of the adventitia was striped off using forceps and the aorta was transferred to a fresh petri dish of sterile phosphate buffered saline. The remainder of the adventitia was stripped away and an incision was made down the length of the aorta. It was then opened and the inner layer was gently scraped off using a scalpel to remove endothelial cells. The tissue was transferred to a 0.85% saline solution containing 2 units/mL penicillin, 2 µg/mL streptomycin, 5 ng/mL of amphotericin and moved to a cell culture hood. The pieces were transferred to a new petri dish containing a few drops of DMEM containing 10% fetal bovine serum (FBS) and finely minced with scalpels into ∼1 mm pieces. The pieces of tissue were then washed out of the petri dish using 100 microliters per aorta of 2 mg/mL collagenase type IV (Life Technologies cat#17104–019) dissolved in 5 mg/mL dispase (Stemcell Technologies, Vancouver, BC; cat# 07193) and placed into 1 well of a 48-well culture plate. Tissue was allowed to digest at 37°C for 1–2 hrs. Following digestion, the collagenase/dispase was inactivated with 5 milliliters of Dulbecco's Modified Eagle Medium (DMEM) containing 10% fetal bovine serum (FBS) and cells were spun at 300×g for 5 minutes. Cells were washed a second time in a similar manner. Cells were then suspended in 2 milliliters of DMEM containing 10% FBS, 100 units/ml penicillin, 100 µg/ml streptomycin, 1% Glutamax (Life Technologies), and 0.1 ng/mL TGF beta. The tissue/cell mixture was distributed into a 48-well gelatin coated culture plate, 1 aorta per well, and placed in a 37°C incubator with 5% CO_2_ and 5% O_2_ to allow cellular outgrowth.

Rat aortic smooth muscle cells were isolated with the same protocol as above with the following modifications: male Sprague-Dawley rats were anesthetized with sodium pentobarbital, 1 milliliter of the collagenase/dispase solution was used for digestion for a full 2 hours at 37°C, and cells from a single aorta were distributed in 6 wells of a 12-well plate. Vascular smooth muscle cells were passed when they reached 50–75% confluence and not used beyond the 4^th^ passage.

### Flow Cytometry Sorting

Bone marrow mononuclear cells were isolated by layering 10 milliliters of fresh human bone marrow onto Ficoll-Paque™ (Stemcell Technologies). Cells were centrifuged at 400×g for 20 minutes at room temperature. The mononuclear cells from the interphase were isolated and washed twice with phosphate buffered saline (PBS), pH 7.4. Flow cytometry was performed using anti-hTAS2R46 (Abcam, Cambridge, MA) as a primary antibody followed by goat anti-rabbit-FITC (Life Technologies) secondary in a total volume of 100 microliters and incubated for 30 minutes at 4°C. Forward and 90 degree side-scatter were used to identity and gate the live cell population prior to sorting on a Becton Dickenson FACSAria I. The resultant flow-sorted cell populations were re-suspended in minimal essential medium (MEM)-alpha contained 20% FBS, 1% penicillin-streptomycin (referred to as MSC growth medium), and incubated at 37°C with 5% O_2_ and 5% CO_2_. Cells were at a concentration of 100,000–300,000 cells per milliliter plated in a T-75 flask (giving a maximal density of 20,000 cells per cm^2^). After 24 hours, non-adhered cells were removed. Adherent cells were washed with PBS and fresh media was added every 3–4 days. Cells were passaged at 80% confluency using 0.05% trypsin, 0.53 mM EDTA (Life Technologies) for 10 minutes at room temperature. Cells were re-plated at a density of 500 cells/cm^2^ and expanded. For co-staining experiments and multiple population sorting, the following antibodies were used: DyLight™ 649 Donkey anti-rabbit IgG (Biolegend, San Diego, CA) as a secondary antibody for rabbit anti-TAS2R46. Other antibodies used included: anti-CD105-fluorescein isothiocyanate (FITC), anti-CD90-FITC, anti-CD73-phycoerythrin (PE), anti-CD31-FITC, anti-CD34-PE, and anti-CD45-FITC with the matching isotype control antibodies; all from BD Pharmingen, (San Jose, CA).

### MSC Differentiation/Staining

For osteogenesis and adipogenesis, cells were plated at 1000 cells/cm^2^ in a 6-well plate and incubated in MSC growth medium for 7 days. For osteogenesis, the cultures were then incubated in 3 mL/well of MEM-alpha supplemented with 10% FBS 100 nM dexamethasone, 0.2 mM ascorbic acid, 10 mM beta-glycerol phosphate, and 1% Penicillin-Streptomycin. Media was changed every 3–4 days for 3 weeks. The cells were fixed with 10% formalin for 20 minutes at room temperature and stained with 2% alizarin Red, pH 4.1 for 20 minutes at room temperature. For adipogenic differentiation, cultures were incubated in 3 mL/well of Iscove's Modified Dulbecco's Media (IMDM, Life Technologies) supplemented with 10% FBS, 10% horse serum, 1 µM dexamethasone, 5 µg/mL human insulin, 12 mM Glutamax (Life Tachnologies), 50 µM indomethacin, 0.5 µM 3-isobutyl-1-methylxanthine, and 1% Penicillin-Streptomycin. Media was changed every 3–4 days for 3 weeks. The cells were fixed with 10% formalin for 20 minutes at room temperature, washed with 60% isopropanol, and stained with 0.6% Oil Red-O for 10 minutes at room temperature. For chondrocyte differentiation, 2×10^5^ cells were pelleted in a 15 mL conical tube at 400×g for 5 minutes. The cells were incubated in 0.5 mL/tube of DMEM-High Glucose supplemented with 1X ITS+1 (Sigma, St. Louis, MO; #12521), 1X linoleic acid with BSA (Sigma, #L9530), 0.1 µM dexamethasone, 50 µg/ml ascorbic acid, 1% Penicillin-Streptomycin, and 10 ng/ml human transforming growth factor-beta_3_ (R&D Systems, Minneapolis, MN). Media was changed every 3–4 days for 3 weeks. The cell pellet was cryopreserved in optimal cutting temperature (OCT) medium (Sakura Finetek U.S.A., Inc.) Six-micron thick frozen sections were mounted on glass slides and fixed in acetone for 5 minutes at room temperature. Slides were dipped in a solution of 50% ethanol containing 1% toluidine blue ten times prior to cover-slipping.

### Immunofluorescence

Cells were plated in glass chamber slides and grown to 75% confluence followed by fixation with acetone for 5 minutes at room temperature. Cells were permeabilized with 0.2% triton X-100 for 15 minutes followed by blocking the cells with 1% bovine serum albumin (BSA) for 1 hour. Primary antibody TAS2R46 (Abcam) was diluted in 0.3% BSA and incubated on the cells for 2 hours at room temperature. Chamber slides were washed with 1x PBS and secondary donkey anti-rabbit Cy3 (Jackson Immunoresearch, West Grove, PA) was diluted in 0.3% BSA and applied for 1 hour. Chambers slides were washed again with 1x PBS. Chambers were removed and the slides were cover-slipped with hard set 4,6-diamidino-2-phenylindole (DAPI) (Vector Labs, Burlingame, CA). Slides were examined by confocal fluorescence microscopy (Olympus BX61, Olympus Optical, Tokyo, Japan).

### Denatonium Labeling and Click-iT Labeling

Stock solutions of 0.5 M denatonium benzoate (Sigma) were prepared in distilled water at 100°C. Denatonium was conjugated to Click-iT succinimidyl ester DIBO alkyne (Invitrogen, Carlsbad, CA) at a 1 denatonium: 0.8 DIBO alkyne ratio, in the presence of 0.1 M NaHCO_3_ (Sigma) at 100°C for 1 hour. Conjugates were kept at 4°C for up to 1 week.

Human mesenchymal stromal cells were plated on 15 mm coverslips and used at 50% confluency. Rat VSMCs were plated on 15 mm coverslips coated with 5 μg/mL fibronectin and allowed to reach 80% confluency. Cells were activated with 1–10 mM denatonium-DIBO conjugate for 2 or 5 minutes on ice or at 37°C. Cells were subsequently fixed with 2% paraformaldehyde (EM Sciences, Hatfield, PA), prior to a copper-free Click-iT reaction using 1 mM Alexa Fluor® 488 azide (Invitrogen) at 37°C for 30 minutes. Labeled cells were mounted in ProLong Gold Antifade (Invitrogen). Due to high autofluorescence in the human MSC, cells were imaged using a Nuance spectral camera (Caliper) with excitation at 450–490 nm and emission collected from 515 nm to 720 nm. Deconvolution of the spectra (Nuance Imaging Software, Cambridge Research & Instrumentation) allowed for separation of autofluorescence and Alexa Fluor® 488 signal. All other cells were imaged using a Zeiss LSM 510 Meta Confocal Microscope with 488 nm excitation and 505–550 nm bandpass emission filter.

### RT PCR

RNA was prepared using Trizol™ according the manufacture's instructions. Forward and reverse primer sequences as listed in Fig. S1. PCR was performed with an annealing temperature of 60°C unless otherwise noted.

### Fluorescent Calcium Assay

Briefly, hMSC were plated at 70% confluence in a 96-well plate in 100 microliters of MSC growth media. The assay was set-up according to the manufacture's protocol, (Fluo-4 Direct™, Invitrogen). Denatonium or other bitter compounds were applied at a range of concentrations and the plate read on a fluorometer every 5 minutes for 30 minutes. The increase in fluorescence was determined by subtracting the background fluorescence (F_0_) from each well pre-denatonium stimulation (F_1_–F_0_/F_0_). For the blocking experiments, 1 microgram of anti-hTAS2R46 (or control rabbit serum) was added to each well 30 minutes prior to denatonium stimulation.

### Calcium Imaging

Cells to be imaged were plated in 8-well chambers 24–48 hours before imaging. For rat aortic smooth muscle cells, the chambers were coated with 5 μg/mL fibronectin. Cells were incubated with 120 nM Fura-2 acetoxy-methylester (Invitrogen) in Tyrode's buffer supplemented with 20 nM glucose and 0.1% BSA (Tyrode's Plus) at room temperature for 15 minutes. Cells were then washed and 300 µl Tyrode's Plus buffer added to each well prior to imaging. Ratiometric imaging was performed on an Olympus iX71, equipped with a Till monochromator to alternate excitation between 360 nm and 385 nm. Emission was collected by an emCCD camera (Andor iXon; Andor™, South Windsor, CT) through a 510/40 nm bandpass filter. During time series acquisition, first 100 μL of buffer was added to the well as a control for stress responses, then 1–10 mM final concentration of denatonium was added. Fura-2 ratio (360/385) images were calculated on pixel-by-pixel basis and an average value from a user defined area within a cell is plotted. Data collection and analysis was performed using AndorIQ software. Physiological temperature was maintained using an objective heater.

### cAMP Assay

Briefly, hMSC were plated at 9600 cells per well in a 96-well plate in 100 microliters of MSC growth media. Denatonium was applied at a concentration of 3 mM followed by incubation for 5 minutes at 37°C. Extracellular cAMP was determined using the ATP Determination Kit from Invitrogen according to the manufacture's instructions.

### ATP Assay

Briefly, hMSC were plated at 70% confluence in a 96-well plate in 100 microliters of MSC growth media. Denatonium was applied at a concentration of 3 mM and ten microliters of supernatant was removed after a 15-minute incubation. Extracellular ATP was determined using the cAMP Chemiluminescent Assay Kit from Invitrogen according to the manufacture's instructions.

### Rat injections

Animals: 6 male Sprague-Dawley rats (Charles River, Wilmington, MA) were used in these studies. Surgical Procedure: Rats were anesthetized with isoflurane (2% mixture in 100% O_2_, Baxter, Deerfield, IL) and given 0.2 mg/kg atropine sulfate via intraperitoneal injection and 2 mg/kg gentamicin sulfate via intramuscular injection (Hospira, Lake Forest, IL) at the time of implantation of an arterial pressure telemeter (model TA11PA-C40, DSI, Saint Paul, MN) and an intravenous catheter (a 7 cm long Silastic tubing with an O.D. of 0.64 mm attached to a 23 gauge Tygon tubing). Approximately 4 cm of the catheter from the telemeter was advanced into the abdominal aorta from a slit at the left femoral artery. The Silastic portion of the intravenous catheter was advanced into the vena cava from a slit at the left femoral vein. The remaining portion of the intravenous catheter was tunneled through the skin and exteriorized at the level of the scapulae though a tether anchor made from a circular piece of Dacron mesh and Silastic tubing (I.D. 6.35 mm, O.D. 12.7 mm). The exteriorized portion of the catheter was threaded through a stainless steel coil that was attached to a swivel that allowed the rat to move freely in its home cage. Further details on the surgical approach have been published previously [29]. Rats were caged individually after surgery and were given 15 mg ampicillin intravenously (Sandoz, Princeton, NJ) and 0.015 mg buprenorphine intravenously (Hospira) for 3 days for post-operative antimicrobial prophylaxis and analgesia, respectively. Rats were allowed to recover for a minimum of 10 days prior to experiment. The i.v. catheter was kept patent by flushing it with 50 U/mL heparin (Sagent, Schaumburg, IL) solution in physiological saline every other day.

Data Acquisition and Analysis: Arterial pressure signal collected by a model RPC1 receiver (DSI) was passed to a PC via a Data Exchange Matrix (DSI) connected to a data acquisition board (DSI) controlled by Dataquest A.R.T. 4.0 software (DSI). Pressure signal was sampled continuously at 500 Hz and binned over 10s for storage. Stored data were exported to a text file for visualization and analysis using Matlab (Mathworks, Natick, MA) and SigmaPlot (Systat Software, Chicago, IL).

Injection of denatonium: A 60 mM solution of denatonium was prepared in physiological saline and filtered through a 0.2 µm filter (Acrodisc PN4192, Pall Corporation, Port Washington, NY) prior to injection. 0.1 mL of this solution was injected into the catheter followed by 0.2 mL of physiological saline to flush the catheter dead space. The whole injection was completed within 30 seconds.

### Statistical Analysis

Comparisons of RQ values for TAS2R46 QRT-PCR results from differentiated MSC (Fig. 2) was performed using Student's t-test comparing each result to that of the undifferentiated cells. To compare the calcium responses of human MSC to different biter compounds (Fig. 4E), a one-way analysis of variance (ANOVA) was performed followed by a Bonferroni post-test. In the rat denatonium injection experiments, the change in arterial pressure after injection of denatonium was analyzed by a one-way repeated measures ANOVA using SigmaStat (Systat Software). A significant ANOVA result was further analyzed by multiple comparisons versus control (arterial pressure 5 minutes prior to injection) using the Holm-Sidak method. Statistical significance was defined at an α-level of 0.05 (Fig. 7C). All other statistical analyses in this work were performed using Student's t-test of the means.

## Supporting Information

Figure S1 Table of primers used in this study. Forward and reverse sequences are given. In the case of Taqman™ primers, the catalog number is listed.
(TIF)

Figure S2 MSC derived from sorted human BMMC based on TAS2R46 receptor expression display cell surface markers prototypical for hMSC. The MSC resulting from the populations shown in Figure 5 were expanded for 2 weeks followed by staining and flow cytometry for the markers indicated above.
(TIF)

Figure S3 Human MSC decrease cAMP in response to denatonium and increase ATP release. (A) 3 week cultured hMSC were treated with 3 mM denatonium for 5 minutes followed by cAMP measurement using a chemiluminescent immunoassay. Results are expressed as a percent of untreated; n = 3 experiments; p-value derived from Student's t-test. (B) Human MSC were treated with 3 mM denatonium for 5 minutes followed by extracellular ATP measurement using a bioluminescence assay. Results are expressed as a percent increase over untreated; n = 3 experiments; p-value derived from Student's t-test.
(TIF)

Figure S4 Murine vascular smooth muscle cells express bitter taste receptors and show a change in morphology. (A) RT-PCR of ortholog bitter taste receptors in cultured murine VSMCs. The + or – indicates the presence of reverse transcriptase (RT). (B) Cultured murine VSMCs were imaged under phase-contrast pre- and post 1-hour treatment with denatonium. Arrows indicate multiple cells that have undergone a change in morphology. Scale bars represent 100 microns.
(TIF)

Figure S5 Human MSC overexpressing hTAS2R46 respond to bioactive peptides in casein hydrosylate. Human MSC transduced with a plasmid expressing TAS2R46 under the control of a CMV promoter and 48 hours after transduction were exposed to increasing amounts of casein hydrosylate (dissolved in PBS). Calcium activity assays were performed at 15 minutes after exposure using the Fluo-4 Direct™ Calcium Assay Kit. The p-values are the result of a Student's t-test, n = 3 experiments.
(TIF)
