# Supplementary figures and images for: Bone Marrow Stromal and Vascular Smooth Muscle Cells Have Chemosensory Capacity via Bitter Taste Receptor Expression
Source: PLoS One. 2013 Mar 8;8(3):e58945. doi: 10.1371/journal.pone.0058945 (PMC3592821; doi:10.1371/journal.pone.0058945)

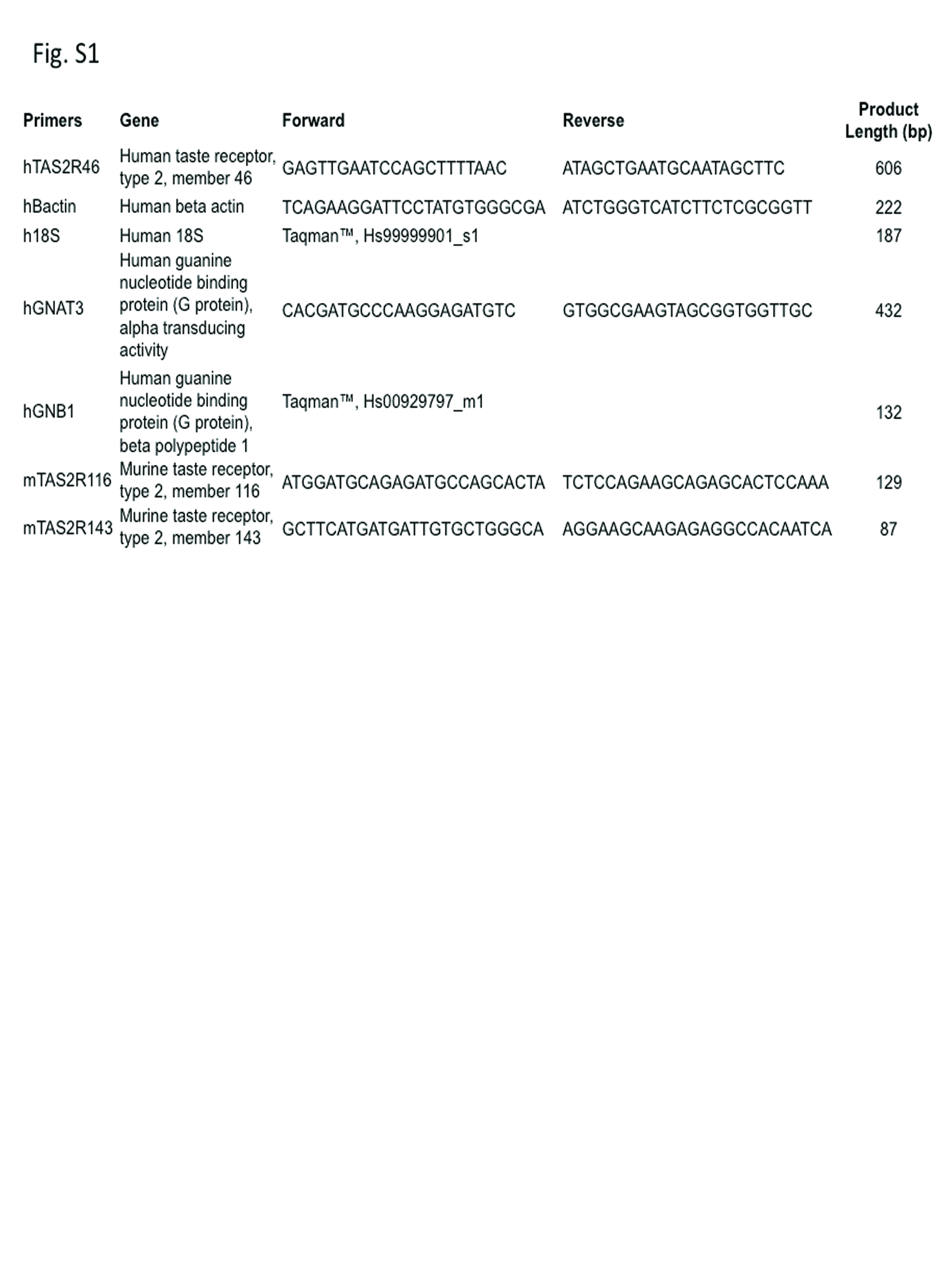

Supplement: Figure S1 — Table of primers used in this study. Forward and reverse sequences are given. In the case of Taqman™ primers, the catalog number is listed. (TIF) [file pone.0058945.s001.tif]

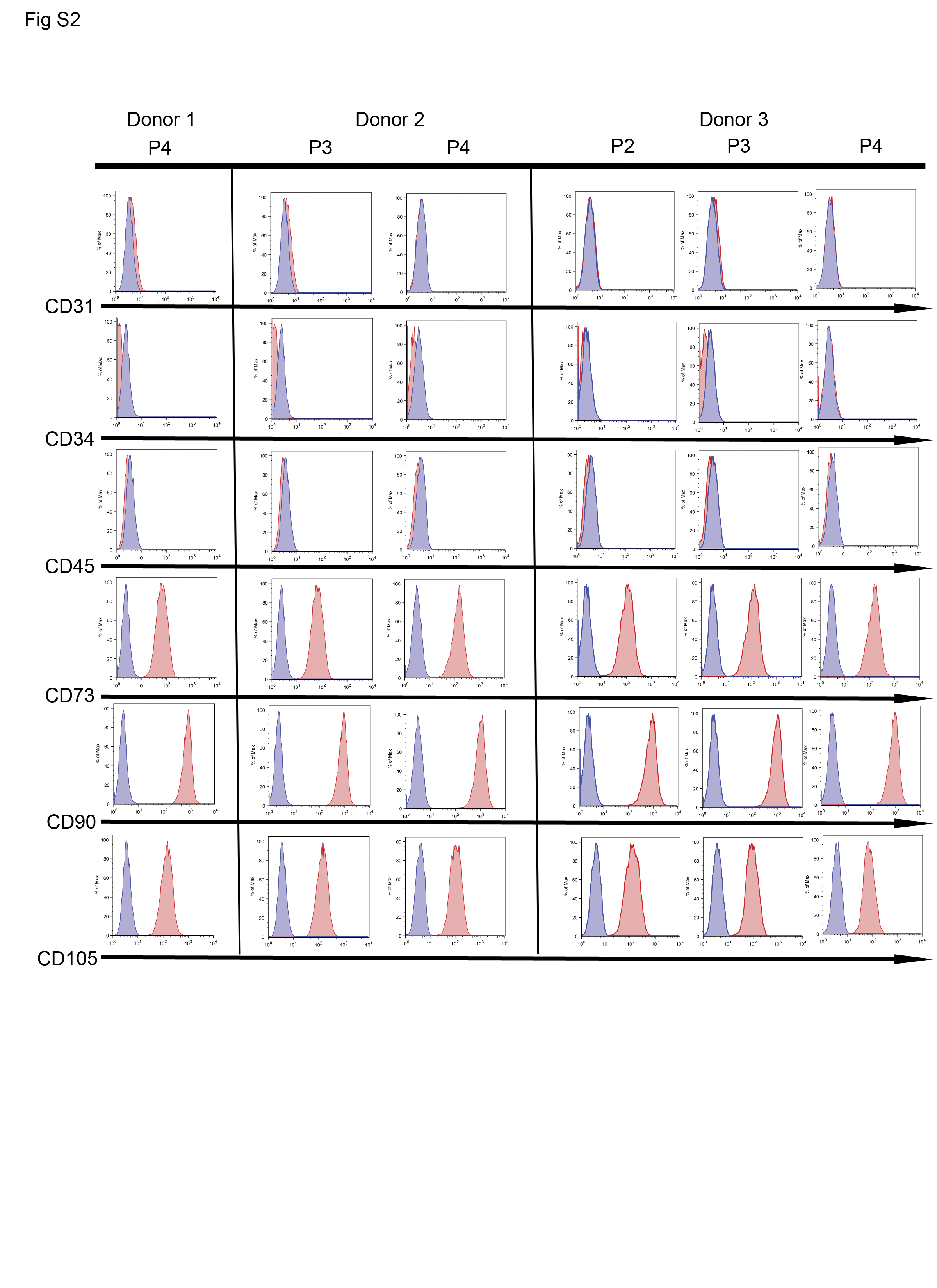

Supplement: Figure S2 — MSC derived from sorted human BMMC based on TAS2R46 receptor expression display cell surface markers prototypical for hMSC. The MSC resulting from the populations shown in Figure 5 were expanded for 2 weeks followed by staining and flow cytometry for the markers indicated above. (TIF) [file pone.0058945.s002.tif]

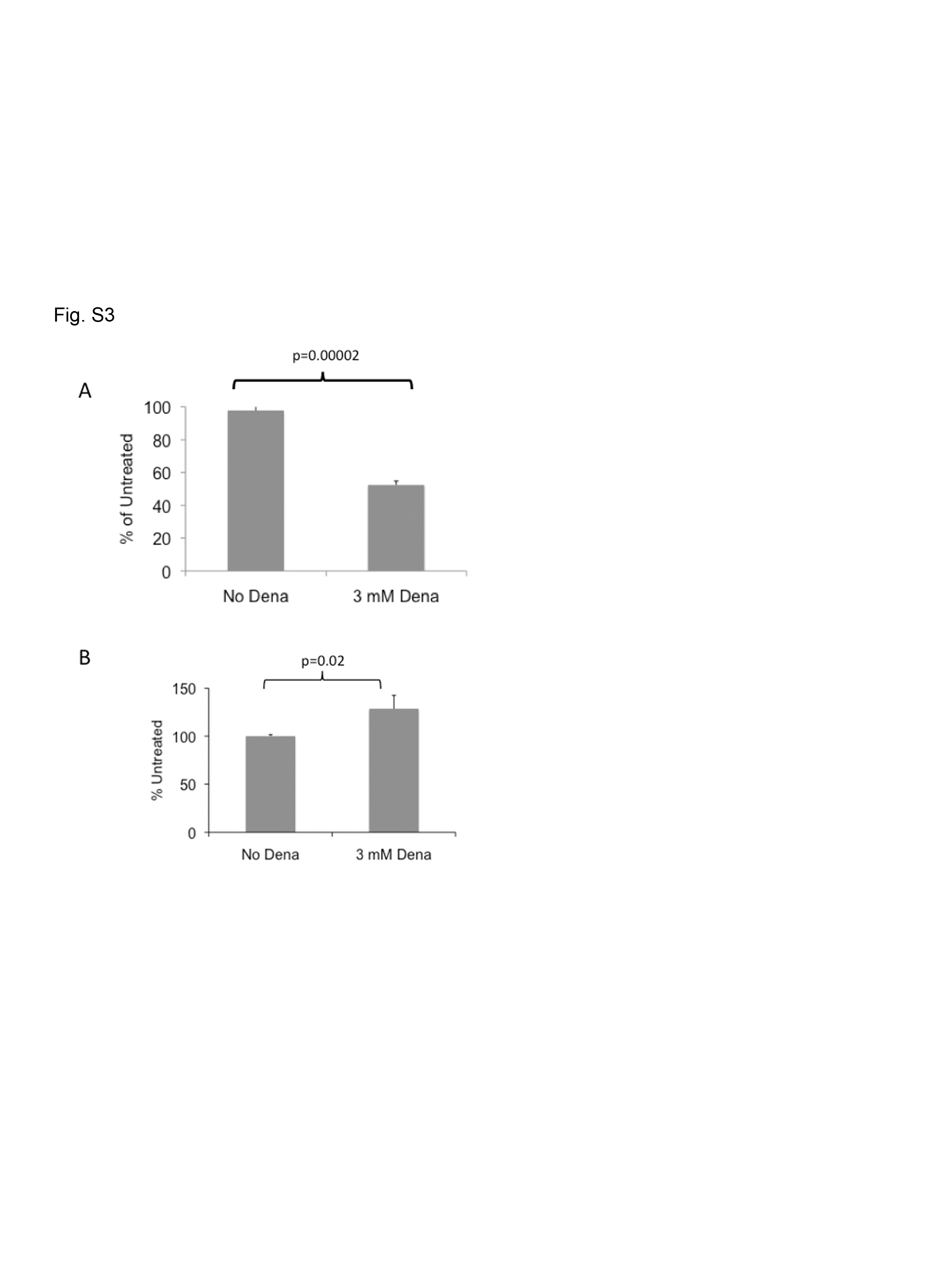

Supplement: Figure S3 — Human MSC decrease cAMP in response to denatonium and increase ATP release. (A) 3 week cultured hMSC were treated with 3 mM denatonium for 5 minutes followed by cAMP measurement using a chemiluminescent immunoassay. Results are expressed as a percent of untreated; n = 3 experiments; p-value derived from Student's t-test. (B) Human MSC were treated with 3 mM denatonium for 5 minutes followed by extracellular ATP measurement using a bioluminescence assay. Results are expressed as a percent increase over untreated; n = 3 experiments; p-value derived from Student's t-test. (TIF) [file pone.0058945.s003.tif]

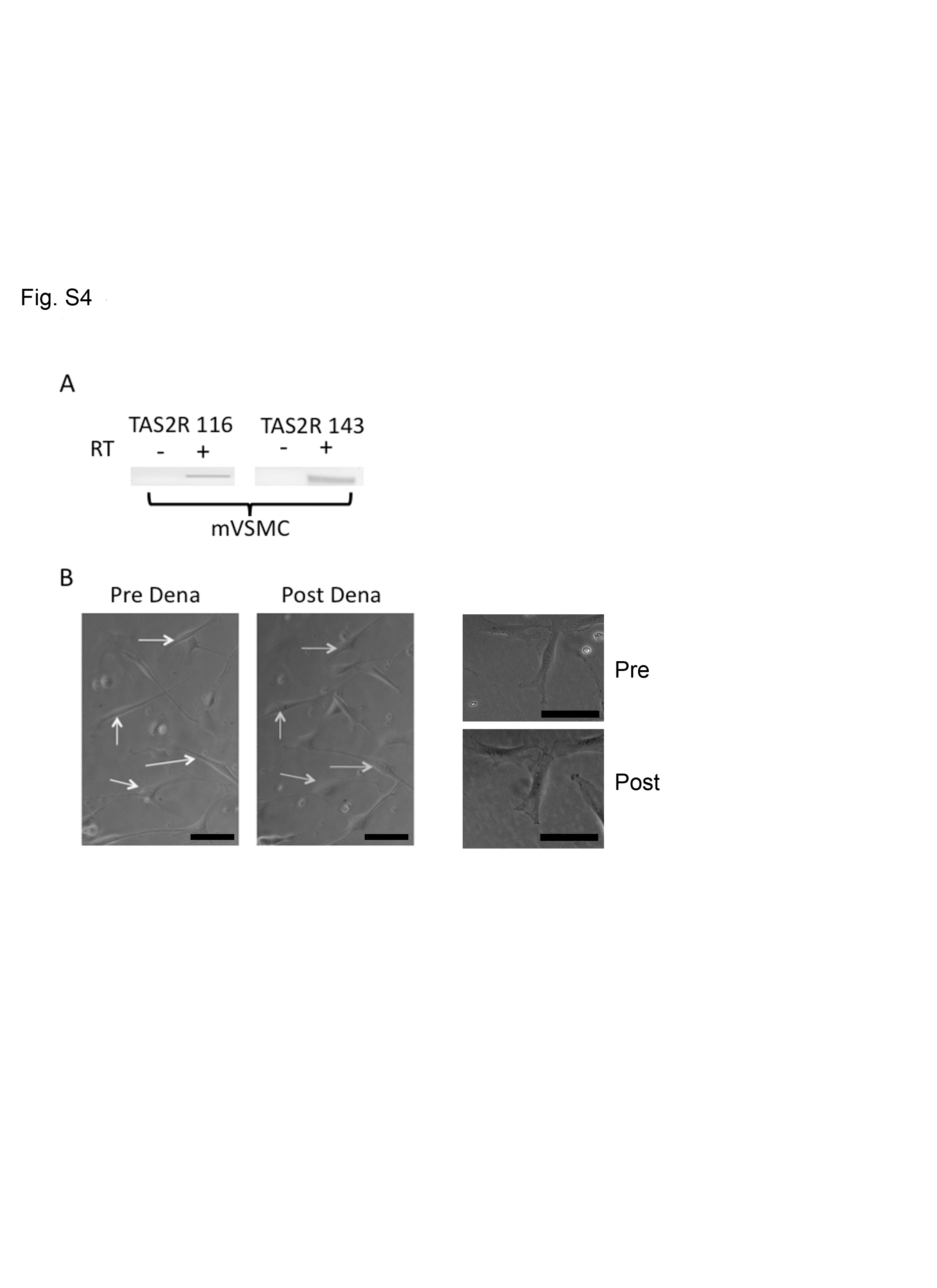

Supplement: Figure S4 — Murine vascular smooth muscle cells express bitter taste receptors and show a change in morphology. (A) RT-PCR of ortholog bitter taste receptors in cultured murine VSMCs. The + or – indicates the presence of reverse transcriptase (RT). (B) Cultured murine VSMCs were imaged under phase-contrast pre- and post 1-hour treatment with denatonium. Arrows indicate multiple cells that have undergone a change in morphology. Scale bars represent 100 microns. (TIF) [file pone.0058945.s004.tif]

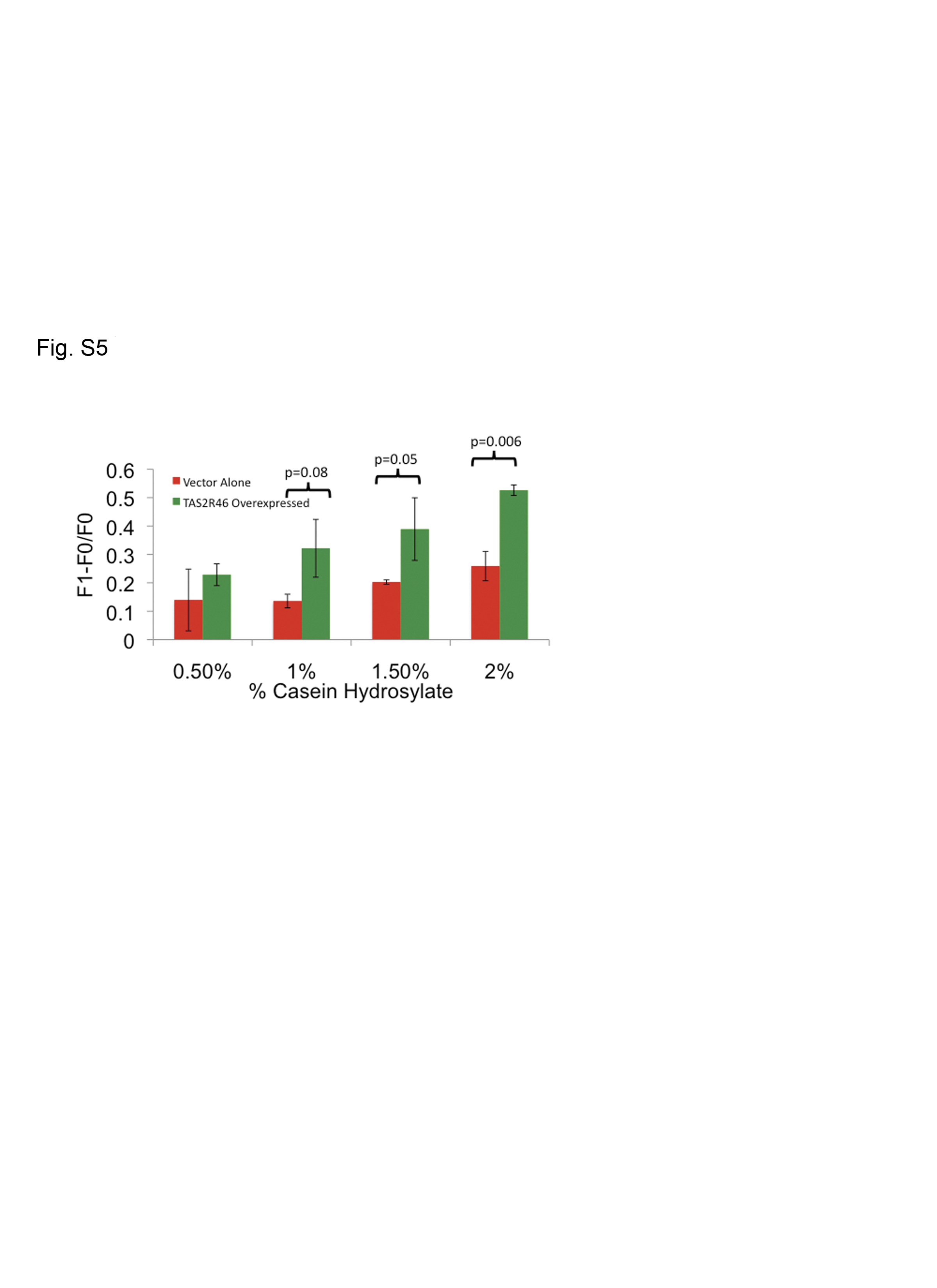

Supplement: Figure S5 — Human MSC overexpressing hTAS2R46 respond to bioactive peptides in casein hydrosylate. Human MSC transduced with a plasmid expressing TAS2R46 under the control of a CMV promoter and 48 hours after transduction were exposed to increasing amounts of casein hydrosylate (dissolved in PBS). Calcium activity assays were performed at 15 minutes after exposure using the Fluo-4 Direct™ Calcium Assay Kit. The p-values are the result of a Student's t-test, n = 3 experiments. (TIF) [file pone.0058945.s005.tif]
